# Optical Coherence Tomography as a Diagnosis-Assisted Tool for Guiding the Treatment of Melasma: A Case Series Study

**DOI:** 10.3390/diagnostics14182083

**Published:** 2024-09-20

**Authors:** Chin-Yi Yang, Ja-Hon Lin, Chien-Ming Chen

**Affiliations:** 1 Department of Dermatology, Chang Gung Memorial Hospital, Linkou Branch, Taoyuan 333, Taiwan; 2 Department of Dermatology, New Taipei Municipal TuCheng Hospital, New Taipei City 236, Taiwan; 3 School of Medicine, College of Medicine, Chang Gung University, Taoyuan 333, Taiwan; 4 Department of Electro-Optical Engineering, National Taipei University of Technology, Taipei 106, Taiwan; 5 Department of Dermatology, Belleesseclinic, Taipei 104, Taiwan

**Keywords:** hyperpigmentation, melisma, optical coherence tomography

## Abstract

**Background/Objectives:** Multiple underlying pathomechanisms may lead to melasma, but there has been no report on the use of optical coherence tomography (OCT) to reveal specific pathomechanisms in individual patients and provide individualized treatments accordingly. Using real-time OCT images, we studied the pathomechanisms of melasma in 12 female patients and the effects of individualized treatments. **Methods:** Patients were divided into good and bad improved groups according to the improvement in hyperpigmentation at month 4. **Results:** In the bad improved group, all melanin or confetti melanin had significantly decreased at month 2 or month 4 while granular melanin ratio at month or month 4 significantly increased, the most parameters of dendritic-sharped cells (DCs) before and after treatment were not significantly different, the collagen area or collagen density at month 4 significantly decreased. In the good improved group, there was slightly low all melanin/confetti melanin at month 4 and high granular melanin at month 4 in comparison to the bad improved group. Moreover, most of the parameters in the DCs at month 4 significantly increased while most parameters in collagen at month 4 significantly decreased. **Conclusions:** OCT is useful in revealing the involved pathomechanisms of melasma in individualized patients. Positive treatment results can be achieved through individualized therapy regimen targeting the pathomechanisms.

## 1. Introduction

Melasma is an acquired hyperpigmentation disorder with the appearance of brown macule, mainly in the centrofacial region, that predominantly affects women with darker skin [1,2,3]. Although the pathogenesis of melasma has not been fully elucidated, some risk factors, including chronic ultraviolet light exposure, genetic predisposition, and female hormone stimulation have been shown to be positively associated with melasma [3,4]. Melasma is not only a melanocyte or melanosome disease, but also a photoaging disease as its histopathologic features overlap with the hallmarks of photoaging skin, such as solar elastosis, altered basement membrane, increased mast cell counts, and increased vascularization [4]. A recent review has proposed five pathomechanisms that may underly melasma: (1) abnormal activation of melanocytes, (2) excessive accumulation of melanin and melanosomes in the dermis and epidermis, (3) increased mast cell count (indicating chronic inflammation) and solar elastosis, (4) altered basement membrane, and (5) increased vascularization [5]. Normal human melanocyte activity has been shown to be regulated by Wnt signaling modulators secreted from fibroblasts, and the Wnt inhibitory factor (WIF)-1 is known to promote melanogenesis [4,6]. Solar elastosis indicates an abnormal accumulation of elastic tissues in the upper and middle dermis associated with chronic UV exposure, and about 83% to 93% melasma sufferers have various degrees of solar elastosis [7,8,9]. Basement membrane disruption occurs in more than 80% of patients with melasma [9]. Pendulous melanocytes, the descent of melanin into the dermis, and a disrupted basement membrane are features of melasma [4].

Optical coherence tomography (OCT) is a non-invasive imaging technique based on the principle of coherent light interference that uses a low-coherent source of light with a relatively long wavelength, typically near-infrared, to image into non-transparent tissues (deeper than is possible with conventional/confocal microscopy) [10,11]. Since its inception in 1991, OCT has undergone many iterations, from time-domain OCT, spectral-domain OCT, to swept-source OCT, gradually improving the resolution and speed of scanning [12]. OCT can provide real-time, two- or three-dimensional (cross-sectional or en face) images with micrometer resolution. OCT has been clinically used in ophthalmology to provide treatment guidance for eye diseases such as glaucoma and retina diseases, but it can also be applied in tumor diagnoses and dermatological examinations [10,13]. In dermatology, OCT can provide images of the skin surface and the underlying epidermis and dermis with depths down to 0.4 to 2.0 mm and an optical resolution of 3 to 15 μm, enabling high resolution at the cellular level and allowing the examination of skin lesions reaching the reticular dermis [10,14,15]. OCT has been used to examine or diagnose various cancerous skin lesions, including basal cell carcinoma, cutaneous squamous cell carcinoma, and actinic keratosis, by revealing certain characteristic features [14,16]. OCT can further be used to follow the patient’s response to treatment in vivo [10]. Other studies have shown that OCT can be used in the differential diagnosis and monitoring of inflammatory dermatosis, such as psoriasis, and in the evaluation of other skin conditions including photoaging, photodamage, scars, folliculitis, onychomycosis, and bullous diseases [17]. As a non-invasive and high-resolution imaging technique, OCT is considered a useful tool for identifying the clinical features and the underlying pathophysiology of various skin lesions.

The treatment of melasma is highly challenging because the outcomes are often inconsistent and relapses frequently occur, probably due to its complex pathogenesis [4]. Identifying the specific pathophysiology of a patient with melasma could help physicians determine the most appropriate therapy or combinatorial therapies and improve outcomes. Here, we used a real-time OCT system as the diagnostic tool to assess the melanocyte activity and basement membrane integrity in a patient, as well as the presence of dermal melanin and/or melanocyte migration to provide an individualized, precise therapy for the treatment of melasma.

## 2. Materials and Methods

### 2.1. Chief Complaints

This case series study was conducted on 12 female patients with melasma (8 cases with skin type III and 4 cases with skin type IV) treated at the Belléesse clinic and New Taipei Municipal Tu Cheng Hospital. Melasma was determined by dermoscopic examination, as described in the previous study [18]. The study protocol was approved by the Institutional Review Board of the Chang-Gung Medical Foundation (approval number: 202101817B0C501). All patients provided signed informed consent to participate prior to the collection of clinical data and images, including consent for publication of the study results. A real-time OCT system was the diagnosis-assisted tool for the treatment of all patients.

### 2.2. Medical History

All patients had no significant history of previous illness.

### 2.3. OCT Assessment

Instruments, acquisition methods, and technical details have been previously described [19]. The full-field (FF)-OCT used in this study was a broadband single-crystal light source (ApolloVue^®^ S100 Image System, Taipei, Taiwan) with a probe used for skin imaging. The light source consisted of a broadband light source with a central wavelength of 750 nm. The B-scan (cross-sectional imaging) scanning area of the OCT measured 500 μm in width × 400 μm in depth. The C-scan (en face imaging) scanning area for the OCT measured 500 μm in width × 500 μm in length × 400 mm in depth. It had an axial resolution of 1.35 μm and a lateral resolution of 1.0 μm.

OCT is an established medical imaging technique in which biological images are captured using light deflected by optical scattering media. It is a non-invasive real-time device with good tissue penetration depth that could potentially be used in the diagnosis of skin lesions [20]. All suspected lesions were identified and cleaned with an alcohol prep pad. Next, the OCT probe was applied perpendicular to the skin. A total of 60 C-scans (continuous lateral scans) of melanin 15 µm above the dermal–epidermal junction of lesional skin [21] and dendritic-shaped cells detected [DCs] at the dermal–epidermal junction of lesional skin [22], and 120 B-scans of collagen detected 100 µm under the dermal–epidermal junction of lesional skin images, were collected per patient. FF-OCT evaluation was performed before treatment and followed up at month 2 and month 4.

For each participant, we employed a computer-aided detection (CADe) system [21,22,23] to automatically extract various statistics representing skin signatures in the detected melanin, DCs, and collagen from the 3D OCT cubes. First, we performed the automatic segmentation of pigments and DCs on average images of en face scans with a thickness of 5 µm. According to previous studies [21,22,23], targets with a diameter greater than 0.5 µm and a brightness exceeding 153 gray scale units were identified as melanin. These were further classified into two types: confetti (diameter > 3.3 µm) and granular (diameter 0.5–3.3 µm). DCs, characterized by elongated bright structures, were detected using the Frangi method [24], with exclusions applied for structures measuring less than 8.5 µm in length or 33.7 µm² in area. Second, collagen detection was also performed on B-scan images. Both the contrast-limited adaptive histogram equalization [25] and the Frangi method [24] were applied to highlight the local contrast and fibrous structure. Furthermore, considering the position of the dermal–epidermal junction (DEJ), the suspected targets with bright filamentous characteristics below them were filtered out. The results were then refined to minimize artifacts caused by oblique lighting, small areas, and variations in image depth.

Earlier studies have shown that melanin appears as hyper-reflective cells with higher intensity on OCT images compared to the surrounding tissues [21], a finding consistent with its appearance on hematoxylin and eosin (H&E) stained slices [23,26]. Additionally, it has been shown that healthy skin, when examined using line-field confocal optical coherence tomography (LC-OCT)—a technique similar to our imaging modality—displays a highly reflective, long fibrous structure (defined as collagen fibers) in the dermis [26].

### 2.4. Treatment Procedure

According to the real-time OCT evaluation, each case received an individualized therapeutic schedule. To treat the increased melanocytes activity, the basement membrane disruption, and hypermelanosis, an individualized treatment was performed depending on their severity using oral tranexamic acid or intradermal tranexamic acid, topical azelaic acid, radiofrequency (RF), a microneedling system (Sylfirm X RF Microneedling system, Roseville, NSW, Australia) with the settings of the PW2 model; 0.6 mm in depth, power level 4–5 (a pulsed-type microneedling RF) (treating interval 3–4 weeks), or a picosecond 1064-nm Nd:YAG laser (Discovery PICO; Quanta System S.p.A, Samarate (VA), Italy), with fluence of 0.5 J/cm^2^, 1000 to 1200 shots of the whole face and 1 pass used.

### 2.5. Outcomes of Treatment

The efficacy of treatment was evaluated at baseline, month 2, and month 4. The documented outcomes included the MASI score, melanin, DCs, and collagen in the lesional skin according to the OCT evaluation.

### 2.6. Statistical Analysis

Continuous variables were expressed as median, mean, minimal, or maximal, and the difference between the two groups was compared using the paired sample *t* test. A 2-sided value of *p* < 0.05 was considered statistically significant. Statistical analyses were performed using SPSS version 25.0 software (SPSS Inc., Chicago, IL, USA).

## 3. Results

A specific OCT examination was used to assist the preoperative diagnosis following the individualized treatment of 12 female patients (median age of 49.5 [41–55] years) with skin type III-IV melasma.

**Figure 1:**
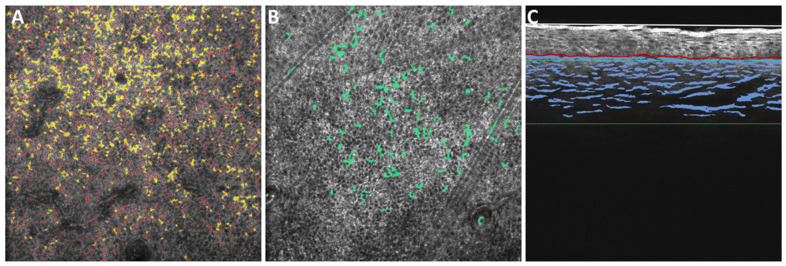
OCT analysis of facial melasma. Cross-section images of each lesion of interest, B-scan or C-scan, were generated and analyzed. In the C-scan images, (**A**) all melanin (brightness level > 153 gray scale and diameter > 0.5 µm, gray color), confetti melanin (area > 8.42 µm^2^ and diameter > 3.3 µm, yellow color), or granular melanin (diameter 0.5–3.2 µm and area = 8.42 µm^2^, red color) 15 µm above the dermal–epidermal junction of lesional skin and (**B**) all DCs in the dermal–epidermal junction of lesional skin were measured. In the B-scan images, (**C**) collagen 100 µm under the dermal–epidermal junction of lesional skin was also measured.

On dermoscopic examination, the lesions in these patients showed diffuse reticular brown pigmentation with concave borders around the openings of sweat glands and hair follicles, manifesting in an ‘exaggerated pseudo-network’ pattern and mimicking the border of a jelly, thus the name ‘jelly sign’ [27], which was determined as melasma. To investigate the underlying pathophysiological changes, a handheld OCT was used. As shown in Figure 1A, OCT examination revealed pendulous melanocytes as well as a remarkably increased accumulation of melanin in all epidermal layers and in the papillary dermis. Moreover, it also showed an increased DC and the degradation of skin collagen (Figure 1B,C). These results indicate the increased activity of melanocytes and basement membrane disruption. However, increased dermal vascularization was not noticed.

**Figure 2:**
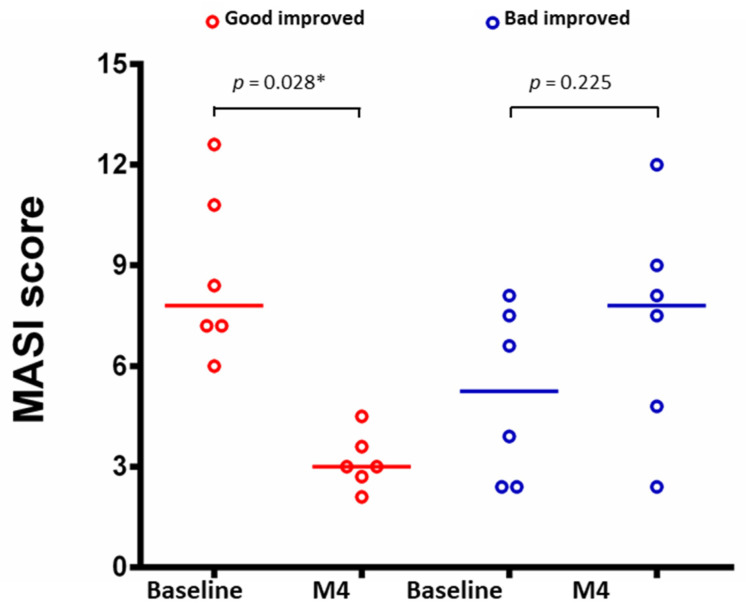
Melasma area and severity index (MASI) scores before and four months after the final treatment. Values were plotted as individuals and median was shown by the horizontal line. * *p* < 0.05.

**Figure 3:**
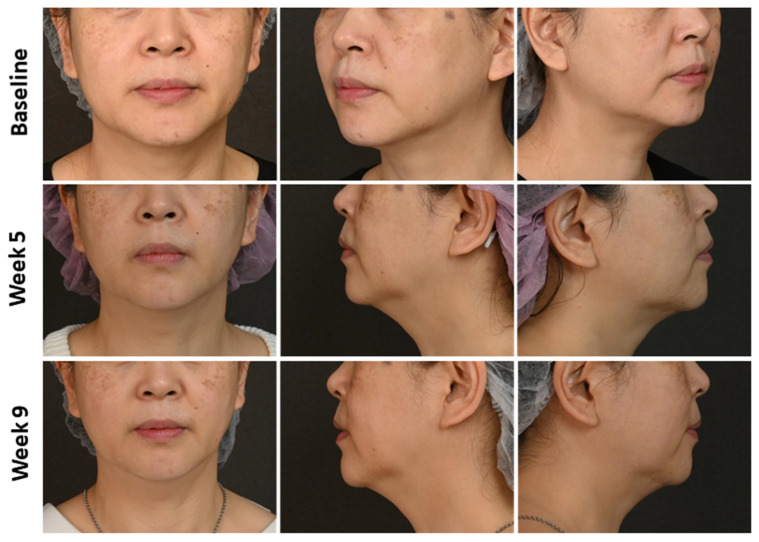
Photographs of facial melasma with good treatment efficacy before and after treatment. A 50-year-old female patient showing melasma in the bilateral cheek.

**Figure 4:**
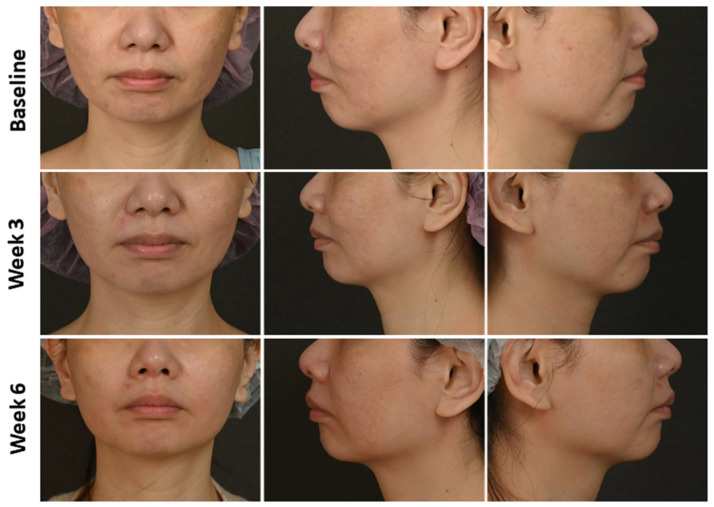
Photographs of facial melasma with bad treatment efficacy before and after treatment. A 43-year-old female patient showing melasma in the bilateral cheek.

According to the severity of real-time OCT examination, these patients received individualized treatment using oral or intradermal tranexamic acid, an RF microneedling system (Sylfirm X RF Microneedling system), or a picosecond 1064-nm Nd:YAG laser. After 4 months of OCT-assisted treatment, patients were divided into good (melasma area and severity index [MASI] [28] significantly decreased [*p* < 0.05, Figure 2] and hyperpigmentation greatly reduced [Figure 3]) and bad (MASI did not significantly decrease [*p* > 0.05, Figure 2] and hyperpigmentation did not reduce [Figure 4]) improved groups. Notably, a significant difference in the MASI scores between the two groups was observed with a slight difference in the photographs, indicating that MASI may be a potential option for predicting response to treatment in patients with melasma. The baseline mean of all melanin size, confetti melanin density, mean confetti melanin size, and confetti melanin ratio (CM and CG) significantly increased in the bad group compared to the good group, while the baseline granular melanin ratio significantly decreased in the bad group when compared with the good group (all *p* < 0.05, Figure S1).

**Figure 5:**
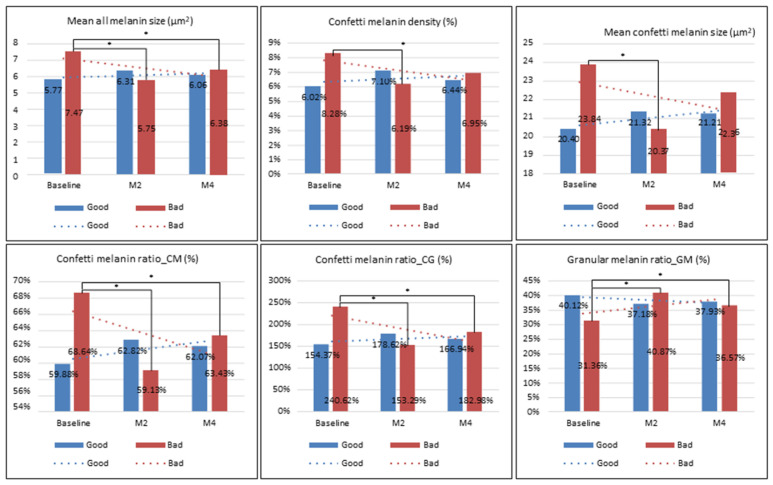
The level of melanin before and after treatment. Mean melanin size, confetti melanin density, mean confetti melanin size, confetti melanin ratio (CM or CG), and granular melanin ratio at baseline, month 2, or month 4 was compared in patients with good or bad treatment efficacy. * *p* < 0.05.

**Figure 6:**
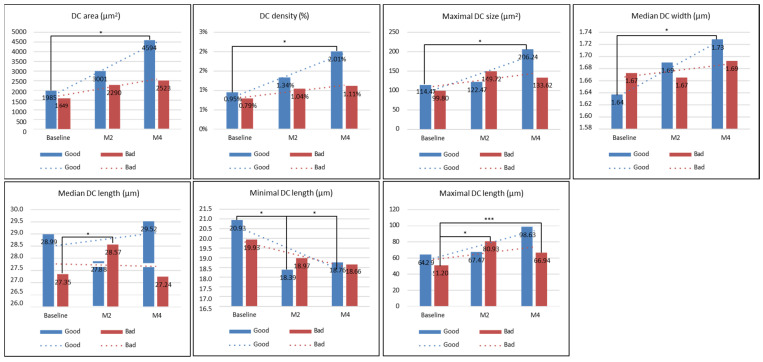
The level of DCs before and after treatment. DC area, DC density, maximal DC size, median DC width, median DC length, minimal DC length, or maximal DC length at baseline, month 2 or month 4 compared in patients with good or bad treatment efficacy. * *p* < 0.05, *** *p* < 0.001.

**Figure 7:**
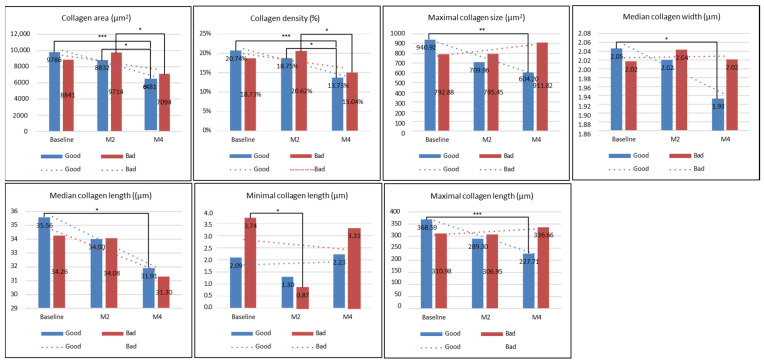
The level of collagen before and after treatment. Collagen area, collagen density, maximal collagen size, median collagen width, median collagen length, minimal collagen length, or maximal collagen length at baseline, month 2, and month 4 was compared in patients with good or bad treatment efficacy. * *p* < 0.05, ** *p* < 0.01, *** *p* < 0.001.

In the bad group, there was a significant decrease in the mean all melanin size, confetti melanin density, mean confetti melanin size, and the confetti melanin ratio (CM and CG) at month 2 or month 4 compared to those at baseline, while there was a significant increase in the granular melanin ratio at month 2 or month 4 compared with those at baseline (all *p* < 0.05, Figure 5). For the DC level, there was no significant difference in the collagen area, DC density, maximal DC size, or minimal DC length at baseline, month 2, and month 4 (all *p* > 0.05), and only the median DC length or maximal DC length at month 2 significantly increased compared with those at baseline (all *p* < 0.05) (Figure 6). The collagen level, collagen area, or collagen density at month 4 significantly decreased compared to those at month 2 (all *p* < 0.05), but there was no significant difference in the maximal collagen size, median collagen width, median collagen length, or maximal collagen length at baseline, month 2, and month 4 (all *p* > 0.05) (Figure 7).

In the good group, there was no significant difference in the mean melanin size, confetti melanin density, mean confetti melanin size, confetti melanin ratio (CM and CG), and granular melanin ratio at month 2 or month 4 compared to those at baseline (all *p* > 0.05, Figure 5). The DC level, DC area, DC density, maximal DC size and median DC width at month 4 significantly increased compared with those at baseline, while the minimal DC length at month 4 significantly decreased compared to those at baseline (all *p* < 0.05, Figure 6). However, the median DC length and maximal DC length at baseline, month 2, and month 4 was not significant different (all *p* > 0.05, Figure 5). The collagen level, collagen area, collagen density, maximal collagen size, median collagen width, median collagen length, and maximal collagen length at month 4 significantly decreased compared to those at baseline (all *p* < 0.05), while the minimal collagen length at baseline, month 2, and month 4 was not significantly different (all *p* < 0.05) (Figure 7).

## 4. Discussion

Due to the multifactorial pathophysiology of melasma, the relapse rate is high and treatment resistance is common. In the present study, we used a real-time OCT system to investigate the pathophysiology of a patient with melasma. Based on the examination results, we provided an individualized, combinatorial therapy targeting the underlying mechanisms, combining tranexamic acid treatment (oral and intradermal injection), azelaic acid, radiofrequency microneedling, and a picosecond laser. To our knowledge, this is the first study that used real-time OCT for treatment guidance in melasma to provide a specialized, multimodal treatment regimen accordingly.

Dermoscopy is considered as the gold standard or first-level screening tool for skin lesions in clinical settings [29]. Dermoscopy is easy to manipulate and does not take long time to perform; however, it can only provide horizontal visualization of superficial skin structures and for most types of common devices used in clinics, the magnification power is limited [14]. Reflectance confocal microscopy (RCM) is another non-invasive imaging technique with high resolution (0.5–1 µm) that enables real-time examination of skin lesions at the cellular level [10], but it is limited in diagnosing equivocal lesions due to its limited imaging depth (200 µm) and may require a combined device (such as OCT) to overcome the limitation [30]. Compared with dermoscopy and RCM, OCT has the depth advantage and enables volumetric visualization of the skin at the microscopic level, making it useful for visualizing structures in the epidermis and upper dermis to provide an accurate diagnosis [14]. OCT skin measurements provide information on skin surface roughness, epidermal thickness, the epidermal–dermal junction, attenuation coefficient, and blood flow [11]. As described previously, melasma is a multifactorial skin lesion, the pathogenesis of which may involve hyperactivity of melanocytes, chronic inflammation, increased vasculature, or a combined mechanism [4]. The pathophysiology presented may vary from patient to patient. In our case, OCT revealed that the melasma resulted from basement membrane disruption and hyperactivity of melanocytes, exhibiting as the accumulation of melanin in the epidermis and dermis, while increased vasculature and vasodilation, which often occur in melasma [5,31], were not observed. Using OCT, we determined the pathogenesis of individual cases of melasma, which traditionally requires invasive histopathological approaches to confirm. The usefulness of OCT in dermatological studies has been further demonstrated in other studies using the advanced type, dynamic OCT (also known as OCT angiography), which can be used to measure vascular characteristics including blood flow and vascular diameter [31,32]. Nevertheless, despite the advantages of high resolution and volumetric visualization, OCT still has some limitations. As indicated by other researchers [14], the diagnostic accuracy of OCT is highly dependent on the skill and experience of the operator, which requires adequate training. In addition, current OCT instruments, especially the advanced ones, are expensive, and it takes time to perform scans. A skilled clinician is also required to interpret the images. In the future, the development of AI-based methods may facilitate the interpretation of OCT images. Moreover, despite the relatively high image resolution compared with conventional non-invasive imaging methods, current OCT instruments cannot achieve the same resolution as histopathology. Future technological advances are required to address these issues.

The wide variety of pathomechanisms of melasma suggests that each mechanism should be addressed in the treatment regimen to maximize the results [5]. Artzi et al. summarized the current treatment modalities for melasma and divided them based on the involved pathomechanistic pathway [5]. To downregulate active melanocytes, treatment agents include hydroquinone, tranexamic acid, methimazole, cysteamine and photobiomodulation; to degrade melanosomes and melanin in the skin, laser (Q-switched, picosecond, ablative and non-ablative fractional laser) and intense pulsed light have been used; to reduce mast cell counts and remodelsolar elastosis, radiofrequency microneedling, tranexamic acid, niacinamide, medicinal plants, calcineurin inhibitors, and corticosteroids have been used; to restore basement membrane integrity, pulsed radiofrequency and heparanase inhibitors have been used; finally, to reduce vascular components, tranexamic acid, pulsed dye laser, and intense pulsed light can be applied. In addition to these treatment modalities, other medical agents have also been proposed. For example, due to safety concerns with hydroquinone, alternative topical depigmenting agents have been considered, including 4-n-butylresorcinol, niacinamide (which also targets mast cell counts and solar elastosis), ascorbic acid, resveratrol, azelaic acid, and kojic acid [4]. Chemical peels are also a well-known technique for treating melasma, especially for those with predominant epidermal pigmentation and short duration [33]. In addition, energy-based devices other than those already mentioned above, such as copper bromide lasers and high-intensity focused ultrasound (HIFU), have also shown positive results in treating melasma [4]. For the melasma in our patients, not all of the described pathomechanisms were involved. Considering the main pathomechanisms in this case were basement membrane disruption, hyperactivity of melanocytes, and hypermelanosis, we used tranexamic acid, which can inhibit the activity of plasmin and tyrosinase [5]; azelaic acid, a depigmenting agent that also has an anti-inflammatory property [4], and radiofrequency microneedling, which can not only restore basement membrane, but also has anti-inflammatory activity and inhibits angiogenesis [5]. We also used picosecond laser, which is effective in degrading melanosomes in the dermis [5]. Due to the positive and satisfactory result achieved in this case, we suggest that for treating melasma, a treatment regimen considering every underlying pathomechanism should be used. Such treatment regimens are individualized, as what works for one patient may not work for another.

In the present study, OCT examination revealed that patients with bad treatment efficacy had a significant decrease in all melanin (size) and confetti melanin (density, size, and ratio) and a significant increase in the granular melanin ratio after treatment. However, these significant changes were not observed in patients with good treatment efficacy, although all melanin and confetti melanin at month 4 were slightly lower in these patients than in patients with bad treatment efficacy. This indicates that OCT examination is a sensitive tool for monitoring post-treatment melanin change in patients with melasma, even in patients with bad treatment efficacy. Moreover, all melanin (size) and confetti melanin (density, size, and ratio) may be potential biomarkers for predicting treatment efficacy in patients with melasma. From this finding, we speculated that patients with bad treatment efficacy may have a significant decrease in post-treatment melanin due to a more severe hyperpigmentation at pre-treatment (lower baseline all melanin, lower baseline confetti melanin, and higher granular melanin ratio), hence this severe hyperpigmentation transforms into a mild hyperpigmentation after treatment (a significant increase in the granular melanin ration).

Furthermore, we found that a significant increase in the dendritic-shaped cell index (DC area, DC density, DC sizem and DC width) at month 4 could be observed in the dermal–epidermal junction (DEJ) of lesional skin in patients with good treatment efficacy after treatment, while the minimal DC length at month 4 significantly increased in these patients. It means our treatment may also induce the activation of epidermal melanocytes after a 4-month treatment, although these patients had a good treatment efficacy. It has been reposted that a Q-switched alexandrite laser removed most melanin from the epidermis; however, numerous activated melanocytes were observed in the subjects on day 7 and continued to be observed until day 28 [34]. Additionally, the presence of bright dendritic cells in melasma after laser treatment showed an early relapse of melasma as these cells correspond to activated melanocytes [35]. Accordingly, we recommended that long-term follow-up is still required for the patients with good treatment efficacy, because the activation of melanocytes may be responsible for the relapse of melasma after treatment.

Obvious collagen loss has been shown in a rat model with melasma [36]. Overall, histologic images reveal unstructured collagen fibers in female patients with melasma [37]. In the present study, the results exhibited that some collagen indexes had significantly decreased at month 4 in patients with good or bad treatment efficacy compared with those at baseline, indicating that our treatment may still lead to a significant collagen loss by month 4. Since human-like collagen repair dressings have moisturizing, film-forming, and repairing effects, and can lighten pigmentation [38], they can also be used to repair damage to the skin barrier caused by melasma and photoelectric therapy. Therefore, we suggest that patients with melasma combine this human-like collagen repair dressing with their individualized therapy.

Recently, a new technique, line-field confocal optical coherence tomography (LC-OCT), was developed and marketed thanks to its extraordinary capacity to acquire high-definition images in vertical and horizontal modes [26]. Combining the principles of OCT and reflectance confocal microscopy (RCM), LC-OCT has a lower penetration depth than conventional OCT, but improved penetration depth compared to RCM. It offers improved image resolution compared to conventional OCT, even though it has lower penetration depth. This tool has been successfully employed to visualize eyelid skin lesions [39] and flat pigmented lesions of the face [40]. In particular, LC-OCT/histopathology in concordance was as high as 92.1% for diagnosis, reaching high standards of diagnostic performance. According to the these findings and the results of our study, a further investigation should aim to determine the efficacy of LC-OCT in guiding the treatment of melasma.

The main limitations of our study were that it was a single-center study with a small sample size. In the future, more randomized control studies across multiple centers and with larger sample sizes need to be conducted to confirm these findings. In addition, melasma was determined by dermoscopic examination, but current OCT instruments cannot achieve the same resolution as histopathology. Although histology is not routinely used in the diagnosis of melasma, further investigations should use histological examination to confirm the efficacy of OCT for guiding the treatment of melasma. Nonetheless, the present study clearly shows that OCT-guided individualized therapy has a definite effect in the treatment of melasma and does not result in relapse at a 4-month follow-up.

## 5. Conclusions

In the treatment of refractory melasma, OCT is a useful tool for identifying the underlying pathomechanisms (melanin, DCs, and collagen) of melasma. Based on the examination results of real-time OCT, a dermatologist can apply the most appropriate individualized treatment for a patient with melasma, which suggests that combinatorial therapy targeting different underlying pathomechanisms can yield positive results.

## Data Availability

The datasets used and/or analyzed during the current study are available from the corresponding author on request.

## Supplementary Materials

The following supporting information can be downloaded at: https://www.mdpi.com/article/10.3390/diagnostics14182083/s1, Figure S1. The level of baseline melanin in patient with good or bad treatment efficacy.
