# Supplementary material for: Optical Coherence Tomography as a Diagnosis-Assisted Tool for Guiding the Treatment of Melasma: A Case Series Study
Source: Diagnostics (Basel). 2024 Sep 20;14(18):2083. doi: 10.3390/diagnostics14182083 (PMC11431355; doi:10.3390/diagnostics14182083)

Optical coherence tomography as a diagnosis-assisted tool for guiding the treatment of melasma: A case series study

Figure S1. The level of baseline melanin in patient with good or bad treatment efficacy. Mean all melanin size, confetti melanin density, mean confetti melanin size, confetti melanin ratio (CM or CG) or granular melanin ratio at baseline was compared in patients with good or bad treatment efficacy.

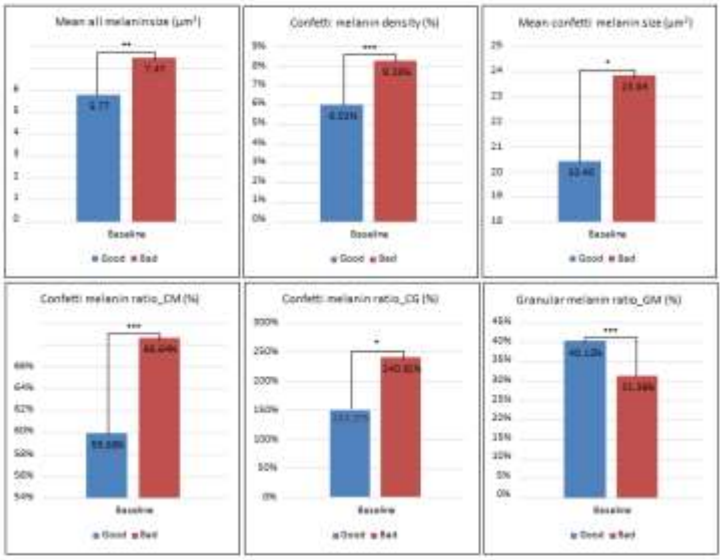

Supplement: Supplementary file 1 [file diagnostics-14-02083-s001.zip › diagnostics-3160050-supplementary.pdf]
